# Patient Education and Levels of Disease‐Specific Information Needs Among Individuals With Oral Epithelial Dysplasia

**DOI:** 10.1111/jop.13642

**Published:** 2025-05-13

**Authors:** Waleed Alamoudi, Abdullah Alsoghier, Richeal Ni Riordain, Stefano Fedele, Stephen Porter

**Affiliations:** ^1^ Faculty of Dentistry King Abdulaziz University Jeddah Saudi Arabia; ^2^ UCL Eastman Dental Institute University College London London UK; ^3^ College of Dentistry King Saud University Riyadh Saudi Arabia; ^4^ Cork University Dental School and Hospital University College Cork Cork Ireland; ^5^ Biomedical Research Centre, NIHR University College London Hospitals London UK

**Keywords:** information needs, mouth precancer, oral dysplasia, patient education, quantitative study

## Abstract

**Background:**

Oral epithelial dysplasia (OED) is a histological diagnosis that carries an increased risk of the individual developing oral squamous cell carcinoma. We assessed the information needs (IN) and explored the sources of education used by individuals with OED using a validated OED‐specific measurement.

**Methods:**

A total of 102 adults with OED from the oral medicine clinic of a dental hospital in Central London were selected using convenience sampling. A cross‐sectional survey was conducted in which participants completed the 33‐item Oral Epithelial Dysplasia Informational Needs Questionnaire (ODIN‐Q), which assessed IN and gathered perspectives on patient education.

**Results:**

Approximately two‐thirds of the participants (*n* = 66, 64%) reported meeting the IN, whereas the remaining participants (*n* = 36, 35%) did not. The mean and median total scores from the questionnaire were 2.43 (± 0.38) and 2.6, respectively, indicating a low sufficient level of IN. Most participants (*n* = 80, 78%) preferred one‐on‐one meetings as the primary mode of obtaining information, followed by written materials (*n* = 64, 62%), audiovisual resources (*n* = 24, 23%), and group discussions (*n* = 8, 0.7%).

**Conclusions:**

Some topics were insufficiently met, necessitating additional educational efforts, such as risk factors and lifestyle modifications, physical and psychological impacts, awareness of potential complications, and seeking medical and psychological support. Sex and degree of dysplasia were associated with the levels of IN. These findings may guide future longitudinal research on OED IN assessment, support the creation of tailored educational tools, and facilitate further evaluation of the psychometric properties of the ODIN‐Q.

## 1 Introduction

Oral epithelial dysplasia (OED) is a histological diagnosis that carries an increased risk of the individual developing oral squamous cell carcinoma (OSCC) [1]. Individuals with moderate‐to‐severe dysplasia are at a significantly elevated risk of oral cancer development, with the likelihood of progression to OSCC increasing 10‐ to 20‐fold compared with those with only cellular atypia or mild dysplasia [2]. Oral potentially malignant disorders (OPMDs) and several risk factors, including alcohol intake, tobacco use, and human papillomavirus (HPV) infection, have been linked to the development of OED [1, 3]. The most frequently affected sites are the tongue, floor of the mouth, and gingiva [2]. The current management strategies for OED include vigilant monitoring and surgical excision [4].

Given the chronic nature of OED, regular and comprehensive patient education (PE) is crucial for achieving favorable long‐term outcomes [5]. The word “doctor” originates from the Latin term “docere,” meaning “to teach,” underscoring the inherent responsibility of physicians to educate patients, their families, and communities [6]. Providing patients with detailed and timely information needs (IN) enhances their understanding of the disease, improves adherence to management plans, and reduces the risk of complications [7]. Furthermore, it elevates patient satisfaction, fosters trust, and enables them to make informed decisions about their health. Effective PE lowers healthcare costs by reducing the frequency of visits, referrals, and resource utilization [7]. However, only 302 articles have explicitly addressed the role of PE in oral and dental disorders and an even smaller number of randomized controlled trials [8, 9]. Moreover, research on the effect of PE on individuals with oral malignancies and OPMDs is limited [10].

The assessment of IN is fundamental for successful PE [11]. IN pertains to the ways in which patients seek and receive knowledge about their disease, diagnosis, treatment, and follow‐up care at the medical centers providing treatment [12]. Previous studies have explored the IN of patients with cancer [13], OSCC [14], and oral precancerous lesions [15]. Despite the widely recognized importance of IN and PE, there is a significant gap regarding the informational and educational needs of patients with chronic dental and oral cavity conditions [8, 9], including OED [5]. The Oral Epithelial Dysplasia Informational Needs Questionnaire (ODIN‐Q) is a recently developed instrument that assesses the informational needs of patients with OED [16], which was validated in 86 patients [16]. The instrument demonstrated excellent internal consistency in the previous study, with a Cronbach's alpha of 0.93 for the overall scale. Test–retest reliability was moderate (*κ* = 0.49–0.53). Moreover, construct validity was supported by a significant, albeit limited, correlation with the Krantz Health Opinion Survey.

We aimed to (1) assess the current levels of IN in adults with OED, (2) explore the clinical factors associated with IN levels, and (3) identify the preferred methods of PE within this population before the development and administration of educational tools.

## 2 Materials and Methods

### 2.1 Study Design and Participant Recruitment

A prospective observational design with quantitative analysis based on questionnaires as part of the PE in OED (EDUCAT‐ED) project was employed. The EDUCAT‐ED project aims to identify the IN of individuals with OED and create tailored educational tools based on their needs and preferences. This study was conducted between March 2023 and December 2024 at the Oral Medicine Unit of the Royal National ENT and Eastman Dental Hospitals at the University College London Hospital (UCLH). Although a larger sample size is necessary to ensure representativeness and a meaningful subgroup analysis, a previous study that assessed IN among patients with oral cavity cancer indicated that a sample size of 92 was required to achieve a power of 0.80 [14].

The present study included a convenience sample of 102 adult volunteers (aged > 18 years), diagnosed with OED based on the 2017 World Health Organization (WHO) diagnostic criteria. Eligible participants included UK residents, proficient in spoken and written English, who could provide informed consent and were without concurrent malignancies or undergoing radiotherapy/chemotherapy to the head, neck, or other regions. All participants confirmed their OED diagnosis with a biopsy procedure conducted at the study site or at external facilities. Consequently, all data were collected during the follow‐up phase of care—no patients were assessed before biopsy or after discharge. After the study was explained, eligible individuals who agreed to participate were provided with a patient information sheet and asked to sign the consent form. The participants were provided with a printed ODIN‐Q to complete after their clinical visit or at home and send it back via post.

### 2.2 Measurements

The ODIN‐Q consists of three sections (Table 1). Section 1 collects sociodemographic details and information on smoking and alcohol intake. Section 2 comprises 33 items that evaluate the adequacy of information provided on various aspects of OED using a 4‐point scale (1 = *none*, 2 = not enough, 3 = *enough*, 4 = *too much*), resulting in a total score ranging from 33 to 132. Section 3 examines patients' preferred methods for receiving IN.

**TABLE 1:** Oral epithelial dysplasia informational needs questionnaire (ODIN‐Q) sections.

Section	Components
Section 1	Seven questions about sociodemographic information, including age, race, ethnic background, level of education, employment status, and smoking and alcohol intake.
Section 2	Thirty‐three questions to assess the knowledge level about the disease, including its diagnostic procedures, therapies, physical and psychosocial impact, and the availability of medical information related to oral epithelial dysplasia.
	Scoring: Questions were assessed using a 4‐point scale (*too much* = 4, *enough* = 3, *not enough* = 2, *none* = 1) and making a total score between 132 and 33, interpreted as the following:
	107–132: Too much information received *(case: highly met IN)*
	81–106: Enough information received *(case: met IN)*
	56–80: Not enough information received *(case: unmet IN)*
	33–55: No information received *(case: highly unmet IN)*
Section 3	One question with multiple options investigating the preferred approach to obtaining information about oral epithelial dysplasia. The options included individual meetings, printed materials, audiovisual resources, and group information sessions.

Abbreviation: IN: information needs.

### 2.3 Analysis of Data and Representation

Microsoft Excel 2022 (version 2410) represented the sociodemographic characteristics, clinical variables, and ODIN‐Q scores. Analyses were performed using SPSS version 27 (IBM manufacturer). A dataset of 102 patients was assessed using descriptive statistics to summarize demographic and clinical variables, and further assessments were done using logistic regression and Spearman's correlation analyses to explore the relationships between these factors and IN. The dependent variable was whether the patient's IN was met, and the independent variables included demographic and clinical characteristics (Table 2). The threshold for statistical significance was set at *p* < 0.05.

**TABLE 2:** The demographic and clinical characteristics of the study participants (*n* = 102).

Variable	Category	Number (%)
Sex	Females	63 (61%)
	Males	39 (38%)
Age, years	20–29	1 (0.98%)
	30–39	2 (1.69%)
	40–49	6 (5.8%)
	50–59	18 (17.64%)
	60–69	35 (34.31%)
	70–79	25 (24.5%)
	80–89	14 (13.72%)
	90–99	1 (0.98%)
Ethnicity	White (British)	50 (49%)
	White (other)	20 (19.6%)
	Asian or Asian British	31 (30.39%)
	Black	1 (0.98%)
Education	College or higher educational degree	59 (57.84%)
	High school diploma or less	41 (40.19%)
	Not reported	2 (1.96%)
Employment	Retired	59 (57.84%)
	Employed (full‐time)	16 (15.68%)
	Employed (part‐time)	7 (6.86%)
	Self‐employed	15 (14.7%)
	Unemployed	3 (2.9%)
	Not reported	2 (1.96%)
Smoking status	Current	14 (13.72%)
	Past (cigarettes)	44 (43.13%)
	Past (smokeless tobacco)	3 (2.94%)
	Never	41 (40.19%)
Alcohol consumption	Current	40 (39.21%)
	Past	21 (20.58%)
	Never	41 (40.19%)
Dysplasia	Mild	74 (43.27%)
Type	Moderate	55 (32.16%)
	Severe	35 (20.46%)
Site	Tongue	51 (42.85%)
	Buccal mucosa	29 (24.36%)
	Gingiva	21 (17.64%)
	Floor of the mouth	8 (6.72%)
	Hard palate	5 (4.2%)
	Lips	3 (2.52%)
	Soft palate	2 (0.84%)
Associated oral disease	Oral lichen planus	86 (63.7%)
	Oral leukoplakia	18 (13.33%)
	HPV‐associated	4 (2.96%)
	Oral submucous fibrosis	3 (2.22%)
	Oral candidiasis	5 (3.7%)
	History of OSCC	19 (14.07%)

Abbreviations: HPV: human papilloma virus; OSCC: oral squamous cell carcinoma.

## 3 Results

**FIGURE 1:**
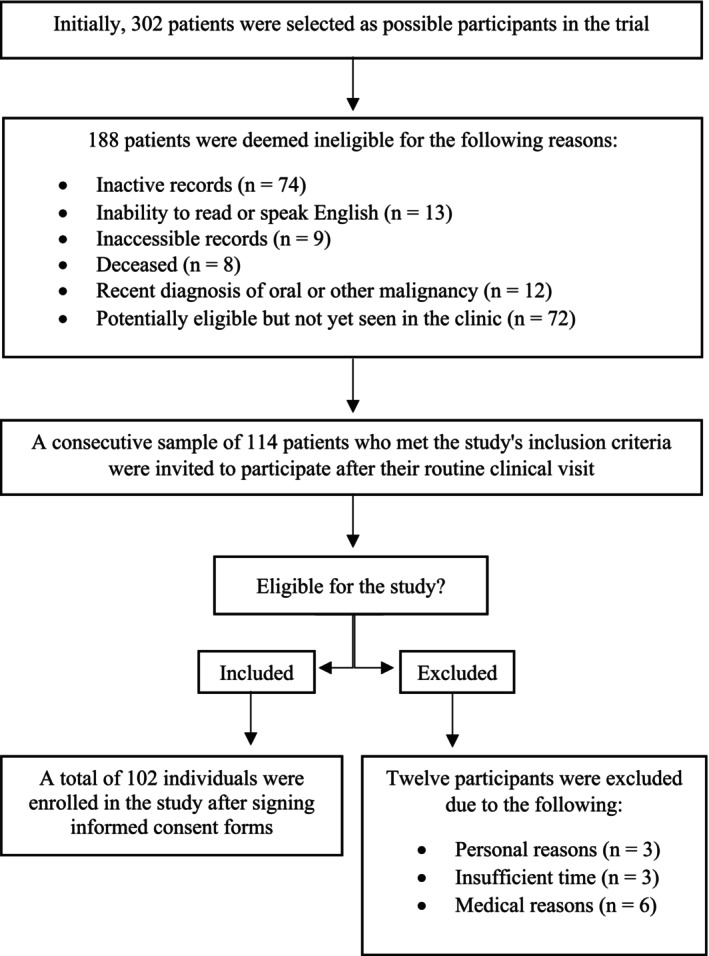
Procedures undertaken to identify and recruit potentially eligible patients.

After a comprehensive investigation of the hospital database, 302 patients were identified as potentially eligible for participation. The step‐by‐step process from identification to final recruitment is shown in Figure 1. The study enrolled 102 participants, and all provided consent by signing a consent form after their scheduled clinical visit.

### 3.1 Participants' Demographic and Clinical Characteristics

Table 2 summarizes the demographic and clinical characteristics of the study participants. The sample was predominantly female (63, 61%), with most participants falling within older age groups; notably, 34.31% were aged 60–69 years and 24.5% were aged 70–79 years. Regarding ethnicity, 49% were White (British), 19.6% White (other), 30.39% Asian or Asian British, and 0.98% Black. The majority (57.84%) of the study participants had a college or higher education degree. Over half of the participants (57.84%) were retired. Lifestyle factors revealed that 13.72% were current smokers, 43.13% reported past cigarette use, 2.94% used smokeless tobacco in the past, and 40.19% never smoked; similarly, 39.21% reported alcohol consumption as a current habit, 20.58% as past, and 40.19% had never taken alcohol.

Analysis of the clinical data and histopathological reports of all the participants revealed 171 biopsies indicating OED. The number of biopsies per participant ranged from one to nine, with an average of 1.69 biopsies per individual. Dysplasia was most often mild (43.27%), with moderate and severe cases accounting for 32.16% and 20.46%, respectively. The total number of clinical sites was 119, as some participants presented with lesions at multiple sites. The most frequent lesion site was the tongue (42.85%), followed by the buccal mucosa (24.36%) and gingiva (17.64%), with other sites less commonly involved. Additionally, 63.7% of patients had oral lichen planus, 13.33% had oral leukoplakia, 2.96% had HPV‐associated lesions, 2.22% had oral submucous fibrosis, 3.7% had oral candidiasis, and 14.07% had a history of OSCC.

### 3.2 Levels of Disease‐Specific IN

According to the predetermined values for the overall ODIN‐Q scores indicated in Table 1, approximately two‐thirds (*n* = 66, 64%) of the participants were satisfied with the amount of IN received. The remaining 36 respondents (35%) stated that their IN was not fulfilled, with 32 of these participants receiving insufficient IN and four respondents receiving no IN on most items. The overall participants' responses to the ODIN‐Q are summarized in Table 3A.

**TABLE 3a:** Participants' responses to the ODIN‐Q (*n* = 102).

ODIN‐Q item	Amount of information received				
	Too much	Enough	Not enough	None	N/A
1. What oral epithelial dysplasia (OED) is?	0 (0%)	86 (84.31%)	8 (7.84%)	8 (7.84%)	0 (0%)
2. How common is it?	4 (3.92%)	56 (54.9%)	23 (22.54%)	19 (18.62%)	0 (0%)
3. What are the risk factors for developing it?	0 (0%)	84 (82.35%)	13 (12.74%)	5 (4.9%)	0 (0%)
4. How it looks in the mouth or lips?	4 (3.92%)	72 (70.58%)	18 (17.64%)	8 (7.84%)	0 (0%)
5. Weather it is contagious or not?	4 (3.92%)	78 (76.47%)	6 (5.88%)	14 (13.72%)	0 (0%)
6. About the role of human papilloma virus.	2 (1.96%)	34 (33.33%)	28 (27.45%)	38 (37.25%)	0 (0%)
7. About the disease grades and risk of developing mouth cancer.	6 (5.88%)	70 (68.62%)	21 (20.58%)	5 (4.9%)	0 (0%)
8. What will happen if I continue to smoke or drink alcohol?	7 (6.86%)	59 (57.84%)	14 (13.72%)	8 (7.84%)	14 (13.72%)
9. What is a safe level of alcohol to drink?	0 (0%)	56 (54.9%)	14 (13.72%)	18 (17.64%)	14 (13.72%)
10. What is likely to happen to OED in the future?	4 (3.92%)	68 (66.66%)	20 (19.6%)	10 (9.8%)	0 (0%)
11. About the screening and early detection.	2 (1.96%)	96 (94.11%)	2 (1.96%)	2 (1.96%)	0 (0%)
12. What are the benefits, risks, how each test works, and the meaning of test results?	0 (0%)	80 (78.43%)	17 (16.66%)	5 (4.9%)	0 (0%)
13. What will happen if it is not treated?	4 (3.92%)	84 (82.35%)	8 (7.84%)	6 (5.88%)	0 (0%)
14. About treatment options, benefits, risks, and how each treatment works?	2 (1.96%)	76 (74.5%)	12 (11.76%)	12 (11.76%)	0 (0%)
15. How the disease/treatment may affect the quality of life?	0 (0%)	58 (56.86%)	32 (31.37%)	12 (11.76%)	0 (0%)
16. About self‐management at home.	0 (0%)	72 (70.58%)	23 (22.54%)	7 (6.86%)	0 (0%)
17. About complementary and alternative medicine (e.g., herbal medicine).	0 (0%)	18 (17.64%)	14 (13.72%)	70 (68.62%)	0 (0%)
18. What are the chances of a cure.	0 (0%)	64 (62.74%)	26 (25.49%)	12 (11.76%)	0 (0%)
19. How frequent and severe are the symptoms (e.g., ulceration, swelling, or bleeding)?	2 (1.96%)	76 (74.5%)	13 (12.74%)	11 (10.78%)	0 (0%)
20. About chances of spreading to adjacent or distant body part?	2 (1.96%)	42 (41.17%)	26 (25.49%)	32 (31.37%)	0 (0%)
21. About the effects of the disease/treatment on daily physical activities (e.g., eating, speaking, or maintenance of oral hygiene).	0 (0%)	72 (70.58%)	20 (19.6%)	10 (9.8%)	0 (0%)
22. About the diet and nutrition.	0 (0%)	54 (52.94%)	32 (31.37%)	16 (15.68%)	0 (0%)
23. About the fear of progression to cancer.	0 (0%)	78 (76.47%)	15 (14.7%)	9 (8.82%)	0 (0%)
24. How to cope with the possible effects of the disease/treatment?	0 (0%)	64 (62.74%)	26 (25.49%)	12 (11.76%)	0 (0%)
25. How the disease/treatment may affect social life (e.g., close relationships, family, and friends)?	0 (0%)	40 (39.21%)	34 (33.33%)	28 (27.45%)	0 (0%)
26. About the experience of your doctor and other healthcare staff.	0 (0%)	94 (92.15%)	8 (7.84%)	0 (0%)	0 (0%)
27. About seeking another professional opinion.	0 (0%)	42 (41.17%)	23 (22.54%)	37 (36.27%)	0 (0%)
28. How to obtain physical support and advice (e.g., who to contact if warning signs appear)?	3 (2.94%)	73 (71.56%)	18 (17.64%)	8 (7.84%)	0 (0%)
29. How to obtain psychological support or advice?	0 (0%)	30 (29.41%)	32 (31.37%)	40 (39.21%)	0 (0%)
30. About community and/patient support groups.	2 (1.96%)	16 (15.68%)	16 (15.68%)	68 (66.66%)	0 (0%)
31. About health promotion (e.g., promoting one's health literacy).	0 (0%)	36 (35.29%)	19 (18.62%)	47 (46.07%)	0 (0%)
32. About the lifestyle adjustment (e.g., tobacco and alcohol cessation and safe sex).	2 (1.96%)	52 (50.98%)	14 (13.72%)	20 (19.6%)	14 (13.72%)
33. About the research and recruitment for clinical trials.	0 (0%)	52 (50.98%)	22 (21.56%)	28 (27.45%)	0 (0%)

Abbreviation: N/A: not applicable.

In addition, the overall analysis of the items of ODIN‐Q revealed a low information sufficiency by a mean and median of 2.43 (± 0.38) and 2.6 out of 4, respectively. Considering the mean, we adopted the following classification: items with mean scores higher than 2.5 are considered “often met,” scores between 2.4 and 2.5 are considered “somewhat met,” and scores below 2.4 are considered “unmet” (Table 3B).

**TABLE 3b:** The mean scores and level of information needs for the ODIN‐Q items.

ODIN‐Q item	Mean score	Information needs^a^		
		Often met	Somewhat met	Unmet
*Information about the disease*				
1. What oral epithelial dysplasia (OED) is?	2.76	✓		
2. How common is it?	2.45		✓	
3. What are the risk factors for developing it?	2.76	✓		
4. How it looks in the mouth or lips?	2.7	✓		
5. Weather it is contagious or not?	2.72	✓		
6. About the role of human papillomavirus.	2			✓
7. About the disease grades and risk of developing mouth cancer.	2.76	✓		
8. What will happen if I continue to smoke or drink alcohol?	2.71	✓		
9. What is a safe level of alcohol to drink?	2.1			✓
10. What is likely to happen to OED in the future?	2.62	✓		
*Information about investigative tests*				
11. About the screening and early detection.	2.96	✓		
12. What are the benefits, risks, how each test works, and the meaning of test results?	2.74	✓		
*Information about treatment*				
13. What will happen if it is not treated?	2.84	✓		
14. About treatment options, benefits, risks, and how each treatment works?	2.66	✓		
15. How the disease/treatment may affect the quality of life?	2.45		✓	
16. About self‐management at home.	2.64	✓		
17. About complementary and alternative medicine (e.g., herbal medicine).	1.49			✓
18. What are the chances of a cure.	2.5		✓	
*Physical aspects*				
19. How frequent and severe are the symptoms (e.g., ulceration, swelling, or bleeding)?	2.68	✓		
20. About chances of spreading to adjacent or distant body part?	2.1			✓
21. About the effects of the disease/treatment on daily physical activities (e.g., eating, speaking, or maintenance of oral hygiene).	2.6	✓		
22. About the diet and nutrition.	2.37			✓
*Psychosocial aspects*				
23. About the fear of progression to cancer.	2.68	✓		
24. How to cope with the possible effects of the disease/treatment?	2.5		✓	
25. How the disease/treatment may affect social life (e.g., close relationships, family, and friends)?	2.11			✓
*Medical system and access to information*				
26. About the experience of your doctor and other healthcare staff.	2.92	✓		
27. About seeking another professional opinion.	2.08			✓
28. How to obtain physical support and advice (e.g., who to contact if warning signs appear)?	2.68	✓		
29. How to obtain psychological support or advice?	1.9			✓
30. About community and/patient support groups.	1.52			✓
31. About health promotion (e.g., promoting one's health literacy).	1.9			✓
32. About the lifestyle adjustment (e.g., tobacco and alcohol cessation and safe sex).	2.07			✓
33. About the research and recruitment for clinical trials.	2.23			✓
Overall mean score	2.43			

^a^ Information needs: often met: mean scores higher than 2.5, somewhat met: mean scores between 2.4 and 2.5, unmet: mean scores below 2.4.

### 3.3 Clinical Variables Influencing the IN

Logistic regression analysis revealed no statistically significant predictors. Backward elimination was used to assess their contribution to predicting the outcome. The complete model initially included all clinical variables. However, there was a trend for sex to be associated with IN, with women showing higher odds of having sufficient IN (odds ratio = 4.459, 95% confidence interval: 0.800–24.852, *p* = 0.088; Table 4). Spearman's correlation analysis revealed a weak relationship between the severity of dysplasia and IN. For mild‐to‐moderate dysplasia, there was a weak negative correlation (*r* = −0.333, *p* < 0.05), indicating that as dysplasia severity increases from mild to moderate, IN may decrease slightly. In contrast, for moderate‐to‐severe dysplasia, a weak positive correlation was found (*r* = 0.327, *p* < 0.05), indicating that as dysplasia severity increases from moderate to severe, IN tends to increase slightly. Both correlations were statistically significant (*p* < 0.05).

**TABLE 4:** Full logistic regression model results.

Variable	Odds ratio (95% CI)	*p*
Age	1.009 (0.952–1.070)	0.760
Smoking status	0.420 (0.075–2.357)	0.325
Alcoholic status	2.727 (0.553–13.453)	0.218
Gender of the participant	4.459 (0.800–24.852)	0.088
Mild dysplasia	0.715 (0.126–4.065)	0.705
Moderate dysplasia	2.225 (0.479–10.344)	0.308
Severe dysplasia	0.756 (0.141–4.059)	0.744
Ethnicity	0.608 (0.354–1.045)	0.702
Education level	0.814 (0.544–1.218)	0.318
Employment status	1.029 (0.541–1.958)	0.930
Constant	3.292 (N/A)	0.672

### 3.4 Preferred Educational Methods for Information Delivery

Participants were allowed to select one or more preferred methods of receiving OED‐specific education, including one‐on‐one meetings (*n* = 80, 78%), written information (printed and online materials) (*n* = 64, 62%), audiovisual resources (videos and podcasts) (*n* = 24, 23%), and group discussions (*n* = 8, < 1%). Among those who preferred one‐on‐one meetings, most preferred receiving information directly from the OED specialists (*n* = 80, 100%). A preference for consultations with general dental practitioners and auxiliary healthcare professionals (e.g., dental assistants) followed this preference (*n* = 17, 21.25%).

## 4 Discussion

In this study, two‐thirds of the participants reported meeting their IN, and one‐third had unmet IN. The current analysis indicates that topics concerning the nature of the disease, investigations, and treatments were well addressed. A possible explanation for this finding is that patients have an established diagnosis in the past and have undergone investigation and therapy; hence, they have adequate IN levels. Other studies have reported that patients with oral precancerous conditions [15], OSCC [14], and other cancer types [13] had high unmet IN related to disease and treatment, especially at the time of diagnosis and at the beginning of therapy. One possible reason for the discrepancy between these findings and ours may be the timing of the assessment. Our study primarily involved patients in the follow‐up phase after receiving an established diagnosis and undergoing investigations and treatment. Thus, their IN may have been addressed during past clinical consultations. In contrast, studies that assessed IN during the initial diagnostic or early treatment phases likely captured higher levels of unmet needs [13, 14]. Differences in study design, patient populations, and the instruments used to measure IN may also contribute to the observed variations. However, these studies had a longitudinal design and reported that the need for disease‐specific IN declined over time after treatment [13, 14].

The findings of this study showed that various topics on IN were somewhat or insufficiently met, including risk factors and lifestyle adjustment (the role of HPV, safe levels of alcohol, smoking cessation, safe sex, diet, and nutrition), clinical characteristics (prevalence, spread to other parts, chances of cure, and alternative medicine), impacts (psychosocial and physical), seeking support (second opinion, psychological, community), and research and clinical trials. Studies on conditions more strongly linked to HPV than to OED have also highlighted a lack of sufficient IN available to patients regarding the role of HPV in mouth malignancies [15, 17]. The present finding indicates a high proportion of participants who exhibited insufficient IN regarding safe levels of alcohol consumption, which can be explained by the fact that 40% of the patients did not drink alcohol. Thus, they may not be aware of the safe or recommended levels for those affected by OED. In addition, the participants in this study reported unmet IN regarding lifestyle adjustments (smoking, alcohol cessation, and safe sex). Previous cancer research has confirmed that patients express the need for individualized and practical information on how lifestyle modifications, including reducing alcohol consumption, quitting smoking, having safe sex, and making dietary changes, could improve their outcomes [18]. Participants in a previous study frequently sought information to support behavioral changes, such as guidance on diet and nutrition [19]. However, this requirement was not met in the current study.

This study showed that participants' IN on the prevalence of OED were somewhat met. The rarity of this disease may explain these findings. In a large‐scale study that surveyed over 1000 patients with cancer in the United Kingdom, it was found that many participants reported unmet IN, specifically requiring more context regarding how common or rare their cancer type was [20]. Our findings also indicated that IN regarding the chances of OED cure were somewhat met. This may be because the prognosis and clinical behavior of OED differ based on the severity and associated oral disease [4]. For example, mild dysplasia can regress without intervention or progress to a greater degree. Therefore, clinicians should demonstrate more educational efforts to their patients regarding all clinical possibilities and the chances of an OED cure in the future. This finding is consistent with a systematic review summarizing 23 years of research on IN in patients with cancer [21], which underscores the fact that patients frequently feel that they do not receive sufficient IN about the broader context of their disease, such as prevalence and prognosis, contributing to confusion and anxiety. Our findings also showed that participants had insufficient IN on whether OED could spread to adjacent or distant body parts. This finding agrees with that of previous cancer studies, where many participants reported the need for more in‐depth information about the likelihood and nature of cancer spread [20, 21].

The findings of this study revealed unmet IN regarding the psychosocial aspects of OED. Evidence supports that unmet IN can result in psychological distress, such as depression and anxiety, disrupting cognitive processes and reducing adherence to health guidelines among patients with cancer [13] and oral precancerous lesions [15]. This association between unmet IN and psychological distress may play a significant role in the findings of previous research that identified high levels of psychological disorders in individuals with OPMDs [22] and OED [23] and those at an elevated risk of developing OSCC [24]. However, these results should not be interpreted to mean that met IN decreases distress associated with cancerous or potentially cancerous conditions. Since the current study did not measure patients' actual knowledge, it is possible that highly distressed patients are informed but continue to express a desire for more information.

The present analysis showed that the level of IN on complementary and alternative medicines was insufficient. However, in a large European sample of over 900 cancer patients, approximately 35.9% used some form of complementary medicine. Yet, many felt that they lacked reliable information from their oncology team and expressed confusion about how to safely combine it with standard treatments and where to find reputable sources of guidance [25].

In the present study, we observed a positive relationship between sex and met IN, with females having higher odds of sufficient IN than males. However, it is important to note that this association was not statistically significant. This trend aligns with previous research suggesting that women are generally more proactive in seeking health information, often using multiple sources such as healthcare providers, online resources, and family or friends [26]. The current analysis also revealed a weak relationship between the degree of dysplasia and IN. Specifically, weak negative and weak positive correlations were observed for mild‐to‐moderate and moderate‐to‐severe dysplasia, respectively. These results are different from those of previous cancer research, showing that patients in the early stages of the disease experience higher IN than those with advanced disease [13]. Similar studies have identified significant correlations with other factors, including younger age [27], varying educational levels [14, 28], ethnic background, and unemployment status [28]. Other studies have noted an association between sufficient IN and current [15] and previous [14] alcohol consumption. Correlations between clinical symptoms, no history of cancer [15], oral conditions, and diagnostic time [14] have also been noted.

In this study, 78% of the participants preferred one‐on‐one meetings as their primary mode of receiving IN, especially from OED specialists, with 62% preferring printed materials and 23% preferring audiovisual resources. A systematic review of patients with cancer reinforces this observation, revealing that healthcare professionals are consistently identified as the primary source of information, followed by printed informational materials [21]. Our study also indicates that patients with OED seek online health information to satisfy their IN; however, the quality of the available online written [29] and audiovisual [30] information about OED remains poor despite 5 years of analysis.

To our knowledge, this study is the first to use a validated OED‐specific instrument to assess IN in individuals with OED. Similar studies on oral cancer [14] and precancerous oral diseases [15] employed generic tools. For instance, Chen et al. used the Cancer Needs Questionnaire Short Form (CNQ‐SF) and Karnofsky's Performance Status Index [14], whereas Lin et al. used the CNQ‐SF, State Anxiety Inventory, and Attitudinal Oral Cancer Scale [15]. These studies focused on patients' IN during the diagnostic and treatment phases, whereas the current study addresses various other aspects (e.g., posttreatment impacts, medical system challenges, and sources of IN). Furthermore, research of this kind, which is integrated with findings from previous studies that have predominantly focused on the active phase of care, could guide evidence‐based interventions to meet the IN of individuals with OED or OSCC. This study provided baseline data for the EDUCAT‐ED project, which can be used in longitudinal research to compare changes in IN after administering educational interventions such as patient information leaflets or videos. These data can also be used as a baseline to further evaluate the psychometric properties of the ODIN‐Q, including its structural validity and responsiveness. By analyzing structural validity (confirmatory factor analysis), it can be verified that the questionnaire items are adequately interrelated to represent the construct, offering more robust evidence of its alignment with patient IN. Similarly, a longitudinal analysis of IN using the ODIN‐Q could enable tracking of changes over time and assess the impact of educational interventions before and after their application (responsiveness).

This study has some limitations. First, our study employed a convenience sample, which lacks random selection, limiting the generalisability of the findings. Consequently, while the statistical tests provide valuable exploratory insights, the conclusions drawn from these analyses should be interpreted considering the evidence from available clinical studies. Second, the findings may not fully reflect the experiences of populations in different contexts because the sample was derived from a single dental hospital in the United Kingdom. Third, the recruitment of participants was conducted at a single point in time. Therefore, it is recommended that longitudinal assessments of patients' needs and information sources be conducted. Researchers are encouraged to assess IN from the time of diagnosis and monitor these needs throughout the disease course to capture changes in IN and educational preferences. Fourth, self‐reported measures—including the ODIN‐Q with its Likert‐scale items—may introduce response bias. The fact that only closed‐ended questions were used could be a drawback of this study, with options such as “too much/enough/insufficient” used for assessing the IN. This format may have allowed participants to guess the correct answers, potentially influencing the accuracy of the results. Future studies might benefit from incorporating a mix of open‐ and close‐ended questions to capture a more nuanced understanding of participants' needs and reduce the likelihood of guessing.

In conclusion, although most patients possessed sufficient IN, specific essential topics require more educational attention from clinicians, including identifying the risk factors and lifestyle modifications (e.g., tobacco and alcohol consumption, the role of HPV, dietary changes), clinical characteristics (e.g., the possibility of spread, the chance of a cure, and prevalence, alternative medicine), awareness of potential impacts (e.g., psychosocial and physical), and seeking medical and psychological support (e.g., secondary professional opinions and community support). Participants ranked one‐to‐one meetings with healthcare professionals as their primary source of IN about OED. Although some clinical factors (e.g., sex and degree of dysplasia) appeared to be associated with IN, these relationships require further investigation in more extensive and diverse samples while considering psychosocial and environmental factors. Integrating qualitative methods can provide deeper insights into individual experiences.

## Author Contributions

**Waleed Alamoudi:** writing – original draft, writing – review and editing, conceptualisation, methodology, funding acquisition, project administration, resources, investigation, formal analysis, data curation, and visualization. **Abdullah Alsoghier:** writing – review and editing, conceptualisation. **Richeal Ni Riordain:** writing – review and editing, conceptualisation, methodology, funding acquisition, resources, supervision. **Stefano Fedele:** funding acquisition, project administration, resources, supervision. **Stephen Porter:** writing – review and editing, conceptualisation, methodology, funding acquisition, project administration, resources, investigation, supervision.

## Ethics Statement

Following registration with the University College London Hospitals/University College London (UCLH/UCL) Joint Research Office (JRO), this study was assigned a JRO reference number/EDGE number 153912; IRAS project ID 318039). This study received a favourable opinion on 16 January 2023, from the National Health Service (NHS) Research Ethics Committees (REC) (specifically, the London—Surrey Borders Research Ethics Committee, reference 22/PR/1743) and ethical approval was obtained on January 26, 2023 from the Health Research Authority (HRA) and Health and Care Research Wales (HCRW).

## Consent

Written informed consent was obtained from participants.

## Conflicts of Interest

The authors declare no conflicts of interest.

## Peer Review

The peer review history for this article is available at https://www.webofscience.com/api/gateway/wos/peer‐review/10.1111/jop.13642.

## Data Availability

The data that support the findings of this study are available on request from the corresponding author. The data are not publicly available due to privacy or ethical restrictions.
